# O044. Frequency-dependent habituation deficit of the nociceptive blink reflex in migraine with and without aura

**DOI:** 10.1186/1129-2377-16-S1-A59

**Published:** 2015-09-28

**Authors:** Maria Grazia Anastasio, Armando Perrotta, Gianluca Coppola, Anna Ambrosini, Giorgio Sandrini, Roberto De Icco, Francesco Pierelli

**Affiliations:** 1 Headache Clinic, IRCCS INM Neuromed, Pozzilli, Italy; 2 Department of Medical and Surgical Sciences and Biotechnologies, “Sapienza" University of Rome Polo Pontino, Rome, Italy; 3 IRCCS Neurological National Institute C. Mondino, Pavia, Italy

## Background

The habituation phenomenon is a frequency-dependent form of non-associative learning which reflects the excitability level of both sensory and pain systems. We previously demonstrated a frequency-dependent deficit of habituation of the conventional blink reflex in migraine[1]. We investigated the habituation of the trigeminal nociceptive system by studying the habituation of the late component (R2) of the nociceptive blink reflex (nBR) in a wide range of stimulation frequencies in subjects with migraine without aura (MWoA) and with aura (MWA).

## Methods

We studied, interictally, 25 MWoA and 17 MWA subjects, as well as 20 healthy control subjects. We delivered a series of 26 electrical stimuli, at different and randomly chosen stimulation frequencies (0.05, 0.1, 0.2, 0.3, 0.5, and 1Hz), subsequently subdivided into five consecutive blocks of five averaged and rectified responses for each stimulation frequency. Habituation was measured as the percentage decrease of the mean area under the curve of the R2 component across the blocks.

## Results

A significant habituation deficit in the R2 component of the nBR was diffusely found at higher (1Hz and 0.5Hz) and intermediate (0.3 and 0.2Hz) frequencies in MWoA when compared to controls. MWA showed a significant habituation deficit at intermediate (0.3 and 0.2Hz) frequencies when compared to controls. No differences in habituation rate were found at lower (0.1 and 0.05Hz) frequencies between patients and controls.

## Conclusions

A frequency-dependent habituation deficit in trigeminal nociception was clearly detected in MWoA at higher and intermediate frequencies. It indicates a wide abnormal processing of painful stimuli at the trigeminal level during the interictal period. On the contrary, MWA showed a clear habituation deficit at intermediate (0.3 and 0.2Hz) frequencies, only revealing a less impaired trigeminal nociceptive excitability during the interictal period. Our data provide further evidence for functional differences between MWoA and MWA.

Written informed consent to publication was obtained from the patient(s).
